# Molecular Survey of Viral and Bacterial Causes of Childhood Diarrhea in Khartoum State, Sudan

**DOI:** 10.3389/fmicb.2018.00112

**Published:** 2018-02-12

**Authors:** Mosab A. Adam, Ji Wang, Khalid-A. Enan, Hongwei Shen, Hao Wang, Abdel R. El Hussein, Azza B. Musa, Isam M. Khidir, Xuejun Ma

**Affiliations:** ^1^National Institute for Viral Disease Control and Prevention, Chinese Center for Disease Control and Prevention, China; ^2^Department of Virology, Ministry of Higher Education and Scientific Research, Khartoum, Sudan; ^3^Futian District Center for Disease-Control and Prevention, Shenzhen, China; ^4^Department of Microbiology and Parasitology, Faculty of Medicine, University of Khartoum, Khartoum, Sudan

**Keywords:** detection, multiple pathogens, diarrhea, phylogenetic tree, Sudan

## Abstract

Diarrheal disease is a major public health problem for children in developing countries. Knowledge of etiology that causes diarrheal illness is essential to implement public health measures to prevent and control this disease. Published studies regarding the situation of childhood diarrhea in Sudan is scanty. This study aims to investigate viral and bacterial etiology and related clinical and epidemiological factors in children with acute diarrhea in Khartoum State, Sudan. A total of 437 fecal samples were collected from hospitalized children <5 years old with acute diarrhea, viral and bacterial pathogens were investigated by using two-tube multiplex RT-PCR. The genotypes of adenovirus and bocavirus were determined by sequencing. Viral diarrhea was identified in 79 cases (62 single and 17 co-infections) (18%), and bacterial diarrhea in 49 cases (37 single and 12 co-infections) (11.2%). Mixed infections in both groups totaled 19 samples (4.3%) with more than one pathogen, they were viral co-infections (*n* = 7, 36.8%) bacterial co-infections (*n* = 2, 10.5%) and viral bacterial co-infection (*n* = 10, 52.6%). Rotavirus (10.2%) was predominantly detected, followed by norovirus G2 (4.0%), adenovirus (1.6%), bocavirus (1%), and norovirus G1 (0.9%). Infection with astrovirus was not detected in this study. The Shigella –Enteroinvasive *E.coli* (EIEC) (8.9%) was the predominantly found bacterial pathogen, followed by *Vibrio parahaemolyticus* (0.9%), enterohaemorrhagic *E.coli* (EHEC) –Enteropathogenic *E. coli* (EPEC) (0.6%) and *Salmonella enteritidis* (0.6%). *V. cholerae, Yersinia enterocolitica* and *Campylobacter jejuni* were not detected in this study. The phylogenetic tree identified adenovirus belonged to genotype 41 and bocavirus belonged to two different clades within human bocavirus 1. Our findings represent the first report that adenovirus 41 is a cause of diarrhea in Sudan and that human bocavirus 1 is the principal bocavirus strain circulating in Sudan. In conclusion, this is the first comprehensive report to elaborate the pathogen spectrum associated with childhood diarrhea in Khartoum State, Sudan. The results obtained in the present study highlighted the current epidemic situation, the diverse pathogens related to childhood diarrhea, and the importance and the urgency of taking appropriate intervention measures in Khartoum State, Sudan.

## Introduction

Diarrhea has been defined as the passing of watery stool more than three times in 24 h, usually caused by consumption of contaminated food or drinks containing any of various pathogens (Vargas et al., 2004). Diarrhea remains one of the leading causes of morbidity and mortality in children in Africa (Ngosso et al., 2015). Diarrhea can be caused by viral, bacterial, protozoan, or helminth and fungal pathogens. It can also be caused by sensitivity to certain types of food or drugs and sometimes by stress (Vargas et al., 2004; Ngosso et al., 2015). Annually, more than 1 billion diarrhea episodes and approximately 2.5 million deaths occur in children under 5 years of age in developing countries (Vargas et al., 2004; Chen et al., 2012; Kotloff's et al.,^2013^; Ngosso et al., 2015). In most of these countries, diarrhea is the third most common cause for young children to visit health care centers, but little information is available in those countries regarding the causative agents (Vargas et al., 2004). Viral and bacterial infections are the most common causes of childhood diarrhea, but they cannot be differentiated based on clinical presentation. Hence, in developing countries treatment of diarrhea is mainly based on symptomatic findings (Ali et al., 2005; Wang et al., 2014). Among viral causes, enteric viruses are recognized as the principal etiologic agents for childhood diarrhea. Five kinds of viruses are considered relevant as a cause of gastroenteritis including rotaviruses, adenoviruses, noroviruses, astrovirus, and bocavirus (Silva et al., 2008). Bacterial pathogens come as the second common causes of diarrhea in developing countries, and such bacteria include *Escherichia coli, Shigella* spp., *Campylobacter* spp., *Salmonella* spp., and *Vibrio parahaemolyticus* (Somily et al., 2014; Wang et al., 2014). However, in sporadic cases of adult diarrhea *Vibrio cholerae, Yersinia, Shigella, and Salmonella* spp., are the most common causative agents in developing countries. Poor hygiene and sanitation, limited access to safe drinking water, and many other conditions such as malnutrition which increase the risk of contracting diarrhea are also common in these countries (Bonkoungou et al., 2013). Ministry of Health in Khartoum introduced a live human–attenuated G1 (P8) oral Rota vaccine (Rotarix) and administrated in two doses at approximately 2–6 months of age since 2011, as a strategy to prevent and control diarrheal infection, but still, the rate of infection is high (Alaaeldeen et al., 2015). Published studies regarding the problem of childhood diarrhea in Sudan are scanty, with only two reports had indicated that rotaviruses and diarrheagenic *Escherichia coli* (DEC) were common causes of childhood diarrhea (Mustafa et al., 2012; Elhag et al., 2013). The aim of the present study is to identify the occurrence of viral agents (rotavirus, norovirus G1, norovirus G2, adenovirus, astrovirus, and human bocavirus) and bacterial agents (Shigella, DEC, *Vibrio parahaemolyticus*, Salmonella, Yersinia, *Vibrio cholerae*, and Campylobacter) in children with diarrhea. The study also aims to investigate the association of particular risk factors, such as age and gender of the patient, the use of antibiotic, and clinical features with the occurrence of the enteropathogens detected.

## Methods

This study was a joint study co-conducted at the Central Laboratory, Ministry of Higher Education and Research, Sudan and at the National Institute for Viral Disease Control and Prevention, Chinese Center for Disease Control and Prevention, China (CDC), Beijing, China. A total of 437 fecal samples comprising of 276 boys and 161 girls, whom mostly live in rural area, were collected in a clean, dry plastic container during two different seasons (the hot dry season from April to June, and the rainy season from August to December) in the year 2014 at Khartoum teaching hospitals. The samples were transported in an ice box on the same day of collection to the central laboratory in the Department of Virology, Sudan. The samples were stored at −20°C until analyzed at the beginning of 2015. The frozen samples were then transported on dry ice to CDC, Beijing, China. The children admitted to hospitals had been clinically diagnosed with acute diarrhea ranging 1–4 days of the samples collection. The participants were between ages < 1- ≤ 2 year (403, 92.2%); >2 - ≤ 4 year (32, 7.3%), and >4–5 year. (2, 0.5%). Among them, 421(96.3%) had been vaccinated for rotavirus while 16 (3.7%) not vaccinated. Patient's data were collected through structured questionnaire including season, age, gender, clinical symptoms, Rotavirus vaccination status and antibiotic use. The study was conducted with the approval of the Ethics Committee of Sudan Academy of Sciences, and written informed consents were obtained from the children's parents.

### Nucleic acid extraction

Viral nucleic acids (RNA, DNA) and bacterial DNA were extracted from 200 μl of 10% fecal suspension in phosphate buffered saline. The viral nucleic acid extraction was done by using QIAamp 96 Virus QIAcube HT kit according to the manufacturer's instructions (Qiagen, Germany), while bacterial DNA was extracted using QIAamp® Fast DNA Stool Mini Kit (Qiagen) according to manufacturer's instructions. The extracts intended for both viral and bacterial assays were eluted in 200 μl of RNase-and DNase- free water (Qiagen) and immediately stored at −80°C and −20°C, respectively.

### Primers of multiplex PCR

A total of 13 chimeric primers targeting viral and bacterial genomes used in the previously published study (Wang et al., 2014) were adopted. The chimeric primers consisted of a gene-specific sequence fused at the 5′ end of the universal sequence which ensures the approximate amplification efficiency of all the microorganisms; thus, all of the chimeric primers had similar annealing temperatures to assure the approximate amplification efficiency.

### Multiplex-RT-PCR and PCR

A two-tube multiplex PCR method adopted from a previous study (Wang et al., 2014) was used in this study. Multiplex one-step RT-PCR was done to detect six viruses (rotavirus, norovirus G1, norovirus G2, adenovirus, astrovirus, and human bocavirus) in tube 1. Multiplex PCR was done to detect seven enteric bacteria (Shigella, diarrheagenic *E. coli, Vibrio parahaemolyticus, Salmonella enteritidis, Yersinia enterocolitica, Vibrio cholerae* and *Campylobacter jejuni* in tube 2. as described in the previous study Wang et al., 2014. The multiplex PCR conditions were carried out according to the previous study Wang et al., 2014. The thermal cycling was performed using PCR Amplifier (ThermoElectronCorp.Vantaa, Finland) followed by the detection of amplified DNA products by capillary electrophoresis using QIAxcel and DNA Screening kit, Qiagen).

### Sequence and phylogenetic analysis

The PCR products obtained from adenovirus and bocavirus positive specimens were subjected to Sanger sequencing (Tsing Ke, Beijing, China). The sequences obtained were edited to remove primer sequences and unreadable sequences at the 3′ and 5′ end using Bio Edit software and then compared with reference sequences by searching for closely related reference sequences in the NCBI GenBank database using the BLAST server (http://www.ncbi.nlm.nih.gov/blast). The reference strains from GenBank representing different genotypes were included in the phylogenetic analysis, and all the selected sequences as shown in Supplementary Data Sheets 1, 2) were aligned using MEGA 5. Neighbor-joining (NJ) trees were constructed using the Kimura two-parameter method, and the reliability was assessed by bootstrap resampling 1,000. The nucleotide sequences of diarrheal viruses described in the present study have been deposited in the GenBank database. The accession numbers are shown in Supplementary Data Sheet 3.

## Results

### The detection of viral and bacterial etiologies in fecal samples

The two-tube multiplex PCR using the QIAxcel machine allowed detection of viral diarrhea in 79/437 (18.0%) cases and bacterial diarrhea in 49/437 (11.2%) cases. Mixed infections were detectable in a total of 19/437 (4.3%) cases in both groups (Supplementary Figures 1, 2). As much more samples were collected from boys (276) than from girls (161) in the age group <1- ≤ 2 year. (403, 92.2%), the comparisons between these variables were not appropriate. Of the 437 stool samples tested by two-tube multiplex PCR assay, tube 1assay detected 79 sequences of five types of viruses (single and co-infection) (45 rotaviruses, 4 norovirus G1, 18 norovirus G2, 7 adenoviruses, 5 bocavirus), whereas astrovirus was not detected in this study. Tube 2 assay detected 49 sequences of 6 types of bacteria (single and co-infection) (39 Shigella or EIEC, 4 *V. parahaemolyticus* and 3 EHEC -EPEC, 3 *S. Enteritidis)*, while *V. cholerae, Y. enterocolitica* and *C. jejuni* were not detected in this study.

**Figure 1:**
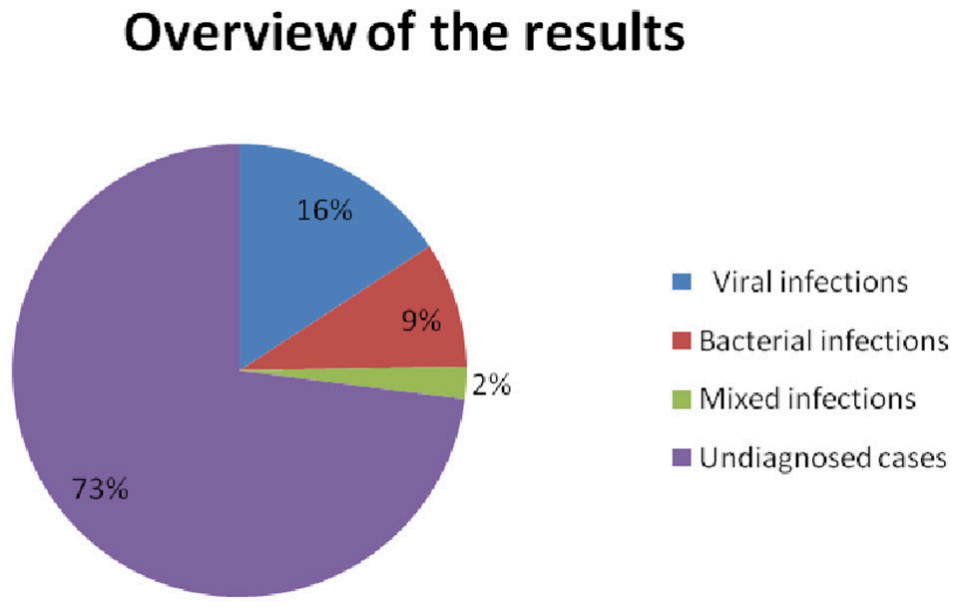
The overview of results that detected 79 viral (69 single −10 co-infections) and 49 bacterial (39 single 10 co-infections) and 319 undiagnosed cases.

Rotavirus was the most frequently detected virus in 10.3% (45/437) samples, followed by norovirus G2 in 4.1% (18/437), adenovirus in 1.6% (7/437), bocavirus in 1.1% (5/437), and norovirus G1 in 0.9% 4/437); (Table 1). The Shigella—EIEC was the most predominantly detected bacteria in 8.9% (39/437) samples, followed by *V. parahaemolyticus* in 0.9% (4/437) and 0.7% (3/437) for each of EHEC - EPEC and *S. enteritidis*. Infection with one type of virus was found in 72 cases (16.5%), while virus co-infection involving rotavirus and norovirusG2 were detected in three cases, rotavirus and adenovirus in two cases, one case of norovirus G2 and adenovirus, and one case of rotavirus and bocavirus (Table 2). Bacterial co-infections were detected in two cases with one for *V. parahaemolyticus* and *S. Enteritidis* and the other case for Shigella–EIEC and EHEC–EPEC (Table 2). As shown in Table 2, mixed infections involving viruses and bacteria were detected in 10 samples (2.2 %). Of the 45 patients with rotavirus infection, 4 came from non-vaccinated, the difference between vaccinated and non-vaccinated patients group was significant (*P-value* 0.049), as shown in Table 3. None viruses or bacteria was detected in 319 diarrheal cases. Figure 1 explains the etiological agents detected in all diarrheal cases. Among all children with diarrheal infections, 68.1 % of whom with viral infections had fever while vomiting occurred in 65.1% of these children. Antibiotics were used for treatment in 80.8% of those children. Of those with bacterial infections, fever and vomiting occurred in 56.8 and 69.8%, respectively. Antibiotics were used in 83.7% of those cases. The incidence of diarrheal cases in the period from August to December (rainy season) was 87.2% while from April to June (hot and dry season) was (12.6%), the incidence of both viral and bacterial infection was high (93.6%, 85.7%, respectively) in the rainy season, but was much less (6.3 and 14.3%, respectively) during the hot and dry season. Table 1 shows the details of infections and the clinical characterization.

**Table 1:** Gender, Age groups, Season, clinical characteristics and Drug by pathogens of diarrheal children.

**Characteristic**											
**Microbes**	**Positive**	**Gender (%)**		**Age (Years) (%)**			**Season (%)**		**Symptoms (%)**		**Drug (%)**
	**Number of patient (%)**	**M**	**F**	**0–2**	**2–4**	**4–5**	**Autumn**	**Summer**	**Vomiting**	**Fever**	**Antibiotic use (%)**
**Viruses**	79 (18)	65.8	34.2	93.7	6.3	0	93.6	6.3	65.1	58.1	80.8
Rota A	45 (10.2)	57.8	42.2	97.8	2.2	0	95.61	4.4	60.6	46.9	80
Noro G2	18 (4)	77.8	22.2	88.9	11.1	0	88.9	11.1	78.6	78.6	89.9
Noro G1	4 (0.9)	50	50	100	0	0	100	0	50	75	75
Adeno	7 (1.6)	85.7	14.3	80	20	0	85.7	14.3	57.1	57.1	85.7
Boca	5 (1.1)	80	20	80	20	0	100	0	80	60	40
Astro	0 (0)	0	0	0	0	0	0	0	0	0	0
**Bacteria**	49 (11.2)	65.3	34.7	95.5	4.5	0	85.7	14.3	69.8	56.8	83.7
Shigella/EIEC^1^	39 (8.9)	64.1	35.9	97.4	2.6	0	82.1	17.9	73	58.8	84.5
*Vibrio Parahaemolyticus*	4 (0.9)	75	25	100	0	0	100	0	50	75	100
EHEC/EPEC^2^^,^^3^	3 (0.7)	66.7	33.3	66.7	33.3	0	100	0	100	0	100
*Salmonella enteritidis*	3 (0.7)	66.7	33.3	100	0	0	10	0	0	100	100
*Yersinia enterocolitica*	0 (0)	0	0	0	0	0	0	0	0	0	0
*Vibrio cholera*	0 (0)	0	0	0	0	0	0	0	0	0	0
*Campylobacter jejuni*	0 (0)	0	0	0	0	0	0	0	0	0	0

1 *Enteroinvasive E. coli (EIEC)*.

2 *Enterohemorrhagic E. coli (EHEC)*.

3 *Enteropathogenic E. coli (EPEC)*.

**Table 2:** Frequency of samples with mixed viral, mixed bacterial, and viral bacterial infections.

**Pathogen**	**No. of co-infections (%)**
rotavirus and norovirus G2	3 (15.8%)
rotavirus and adenovirus	2 (10.5%)
norovirus G2 and adenovirus	1 (5.3%)
rotavirus and bocavirus	1 (5.3%)
*V. parahaemolyticus* and *S. Enteritidis*	1 (5.3%)
shigella-EIEC and EHEC–EPEC	1 (5.3%)
rotavirus and Shigella	4 (21.1%)
rotavirus, adenovirus, *V. parahaemolyticus*, and *S. enteritidis*	1 (5.3%)
norovirus GII and Shigella	1 (5.3%)
norovirus GII and *V. parahaemolyticus*	1(5.3%)
adenovirus, *V. parahaemolyticus*, and Salmonella	1 (5.3%)
adenovirus and *E. coli*	1 (5.3%)
bocavirus and Shigella	1 (5.3%)
Total	19 (100%)

**Table 3:** Cross-tabulation of the frequency of rotavirus infection among rotavirus-vaccinated and non-vaccinated subjects using A chi-square test.

**PCR**		**Vaccinated**	**Not vaccinated**	**Total**	***P*-value**
Rotavirus	Positive	41 (9.7%)	4 (25%)	45 (10.3%)	0.049^*^
	Negative	380 (90.3%)	12 (75%)	392 (89.7%)	
	Total	421 (100%)	16 (100%)	437 (100%)	

* *Means the result is significantly different*.

### Nucleotide sequence and phylogenetic analysis of adenovirus and bocavirus positive samples

**Figure 2:**
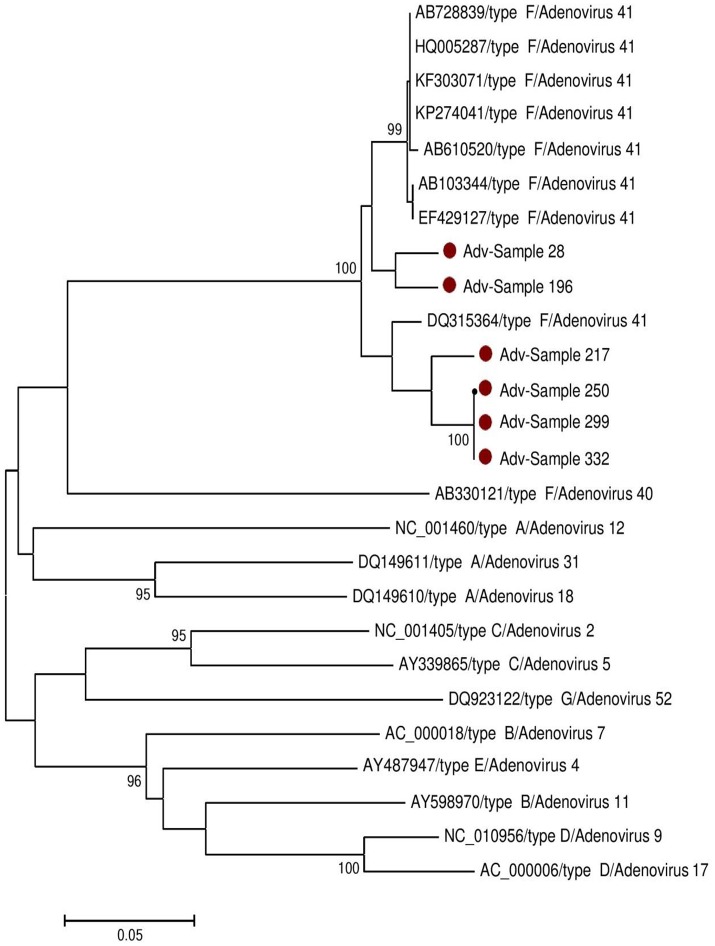
Phylogenetic analysis of Adenovirus sequences isolated in Sudan (28, 196, 217, 250, 299, and 323). The Hexon region was used for genotyping (see Supplementary Data Sheet 2). The Kimura's two-parameter model with 1,000 replications of bootstrap sampling implemented in MEGA 5 was used to analyze the data. Only Bootstrap values >70% are indicated at nodes. Bars show distances. •represent Sudan Adenovirus isolates in this study.

**Figure 3:**
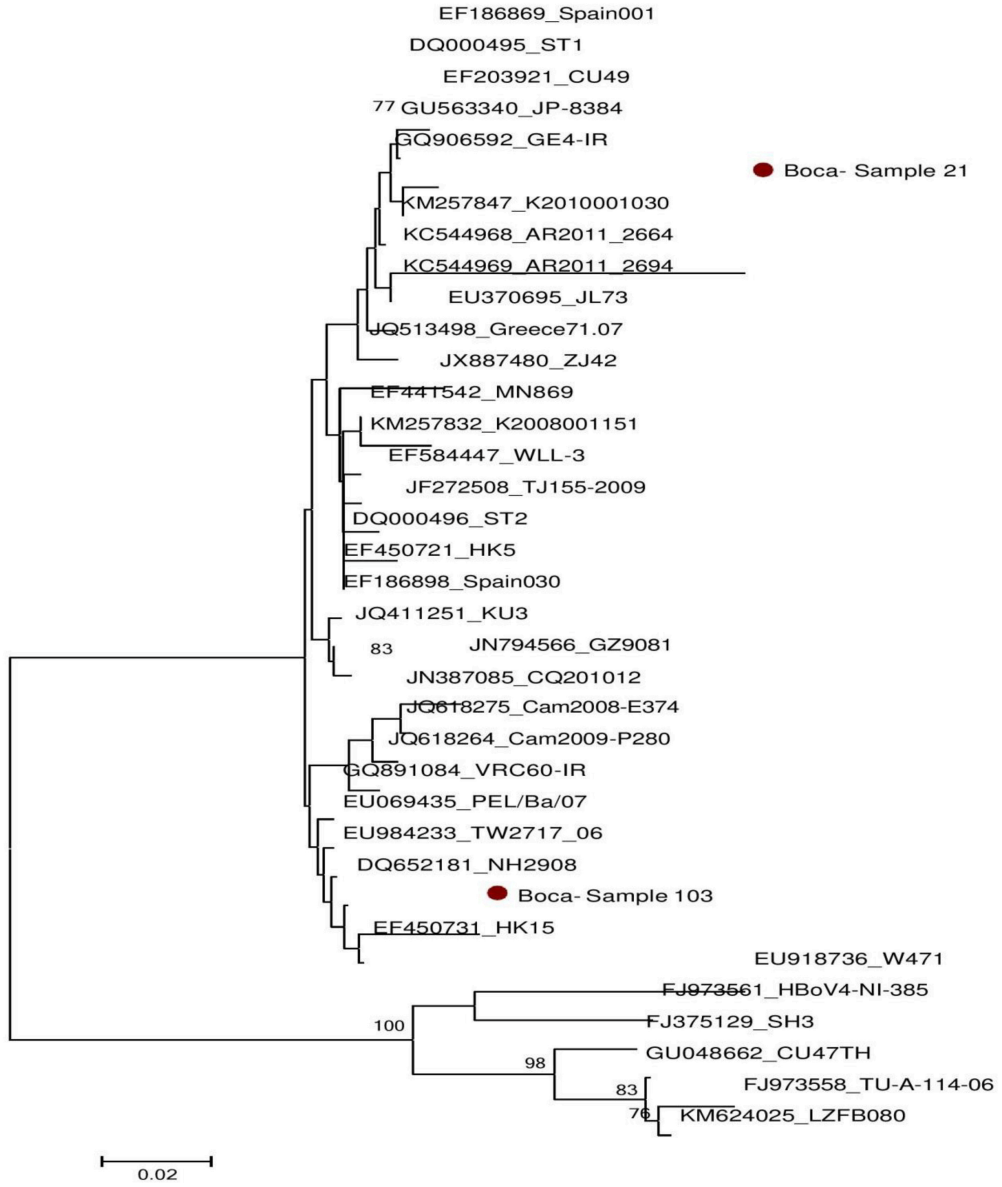
Phylogenetic analysis of Bocavirus sequences isolated in Sudan (21,103). The VP1 region was used for genotyping (see supplementary Data Sheet 3). The Kimura's two-parameter model with 1000 replications of bootstrap sampling implemented in MEGA 5 was used to analyze the data. Only Bootstrap values >70% are indicated at nodes. Bars show distances. •represent Sudan Boca virus isolates in this study.

Successful sequencing of adenovirus-based on hexon gene was done on six samples and bocavirus based on VP1 gene on two samples. The obtained nucleotides sequences were compared with reference sequences published in GenBank (Supplementary Data Sheets 1, 2). Phylogenetic trees were generated for both adenovirus and bocavirus as shown in Figures 2, 3 respectively. Based on nucleotide sequence analysis, all six adenoviruses detected in the present study belonged to adenovirus 41. The phylogenetic tree showed that these strains belonged to two clades related to the strains found worldwide. Samples No 217, 250, 299, and 332 were closely related to the Netherlands strain (Lemiale et al., 2007). While the Sample 28 and 196 were linked to several strains found around the globe (Ireland, Japan, Vietnam, Korea, China, Côte d'Ivoire, USA, and India), as shown in Figure 2. For bocavirus, the nucleotide analysis revealed that the two sequences from samples 21, 103 were related to HbV1. whereas the phylogenetic tree indicated that they were two genetically distant strains. Figure 3 indicated sample 21 was related to several strains around the world (Netherlands, Iran, Japan, Thailand, Sweden, and Spain) and the sample 103 was similar to several strains from other countries (Hong Kong, USA, Taiwan, Italy, Iran, Cambodia, and China).

## Discussion

Diarrheal diseases are the major public health problem for children in Sudan and most developing countries (Bonkoungou et al., 2013; Wang et al., 2014). Knowledge of causative agents that cause diarrheal illness is critical in the implementation of appropriate public health measures to prevent and control these diseases (Youssef et al., 2000). This study was conducted in two seasons (Autumn and Summer) from April to December in 2014, in Khartoum State, Sudan. The aim of this study was to determine some of the viral and bacterial pathogens associated with acute gastroenteritis in stool samples from children <5 years old using a two-tube multiplex PCR assay developed in a previous study (Wang et al., 2014). In this study, the fecal specimens collected from children with diarrhea were positive for viral (18%), and bacterial (11.2%) agents. Five viruses (rotavirus, norovirus G1, norovirus G2, adenovirus, bocavirus) and 6 bacteria species (Shigella, EIEC, *V.parahaemolyticus*, EHEC, and EPEC, *S. Enteritidis*) were detected in this study. Both single and multiple viral or bacterial infections (co-infections) were also detected. The impact of co-infection on clinical severity was not studied for these patients; however, it has been previously reported that no significant differences were observed in the clinical symptoms of patients with co-infection compared with those with single infections (Saikruang et al., 2014). The incidence of diarrhea (27%) caused by pathogens in this study is lower than that obtained in other studies (Elhag et al., 2013; Magzoub et al., 2013; Mustafa et al., 2014; Wang et al., 2014). In Sudan, the positive rate of viral diarrhea cases detected in the present study was 18%, slightly higher than other previous studies (16%) which were conducted to detect rotavirus in Khartoum State and had a similar population in terms of the gender and age exposed to infection (Magzoub et al., 2013). Also, the incidence of our report was higher than another survey in Khartoum State detected rotavirus and adenovirus which was partially similar to our report in the area of research conducted but different in the age groups studied (children and adult) (Elhag et al., 2013). However, we identified new emerging viruses for the first time in Sudan, including norovirus G1, norovirus G2, astrovirus, and bocavirus. Furthermore, we used sensitive automated capillary electrophoresis system in the detection of these pathogens, which may have increased the detection rate of these agents. In this study, rotavirus was the predominant pathogen causing diarrhea in childhood. The finding is similar to the previous study of the Global Enteric Multicenter Study (Kotloff et al., 2013) and other studies in developing countries (Yan et al., 2003; Ali et al., 2005; Bonkoungou et al., 2013; Onori et al., 2014; Wang et al., 2014).

The rotavirus infection was prevalent among vaccinated patients and children under two years. Thus, it may indicate that locally circulating serotypes are not included in the Rotarix vaccine, which consists of just one serotype G1P8 or that the vaccine might have deteriorated during transport or storage. It may also indicate that children in the age group <2 years old may not have completed the vaccine regimen. Similar findings were reported previously in the Central African Republic and Sudan (Banga-Mingo et al., 2014; Alaaeldeen et al., 2015). In our study, the test results indicated that the incidence of norovirus and adenovirus was lower than previous studies in Sudan (Elhag et al., 2013; Mustafa et al., 2013). This difference may reflect differences in sample size, age tested and timing of these studies. Our current study is the first report on the detection of norovirus G1, bocavirus, and astrovirus in Sudan, although the incidences of these viruses were lower than reported from other country (Wang et al., 2014).

The most common serotypes of adenovirus that causes gastroenteritis worldwide are adenovirus 40, and 41 in subgroup F. Sequence analysis of the partial genome of adenovirus in Sudan showed that all the detected strains belonged to adenovirus genotype 41 and these six strains could be further divided into two clades. To our knowledge, this study also represents the first report that adenovirus 41 is one consider as a cause of diarrhea in Sudan. Our study also for the first time describes the primary human bocavirus (HBoV) genotypes circulating in Sudan. The analysis based on VP1 (viral capsid protein) sequences revealed that the viral DNAs belonged to human bocavirus 1 with two different branches. Our findings also suggest that HBoV1 is the main genotype circulating in Sudan.

The incidence of bacterial infection (11.2%) in Khartoum State in this study is lower than that reported in a previous study (19%) in the same area (Mustafa et al., 2012). The differences in the results may be due to differences in the type of stool specimen; sample size tested and different testing methods.

It is noted that no positive samples for *V. cholerae, C. jejuni*, or *Y. enterocolitica* were detected. Shigella-EIEC was the second most common pathogen detected in our study, in line with results obtained by other investigators (Youssef et al., 2000). However, our primer targeted the same gene (ipaH) for detection Shigella and EIEC, so we cannot differentiate between them.

We also found that rotavirus was the most common pathogen involved in co-infections, which is similar to reported results in another study in China (Wang et al., 2014). Our findings may be as a result of rotavirus pathogenesis, which causes enterocyte destruction leading to enhanced opportunistic pathogen infection and this explain that rotavirus was the common pathogen involved in co-infection. Additionally, children at the age <2 year may be subjected to extreme exposure to bacteria such as *E. coli* (Jourdan et al., 1998). Our study also revealed that the incidence of both viral and bacterial infections was much higher in rainy Season (wet) than in summer(dry-hot), in contrast to a study that reported viral infections occurring mainly during the dry season in Burkina Faso (Bonkoungou et al., 2013). The difference could be due to statistical bias because most of our samples were collected during autumn. Nonetheless, this may warrant further research with adequate sample sizes that cover all seasons of a year. In addition, the present study highlighted antibiotic misuse for the treatment of diarrhea, which necessitates improving knowledge about the differentiation between viral and bacterial infections, in addition to developing diagnostic methods in our hospital laboratories. The highest number of positive samples was found in the boys and age group <2 years (Table 1) due to the majority of our samples collected from boys and the age group <1- ≤ 2 year. The reason for the bias in the numbers of children with diarrhea (boys of <1- ≤ 2 year.) admitted to hospitals is not clear and need to be further investigated to determine whether or not it is the pattern of childhood diarrhea in Sudan.

It is of note that no pathogens were detected in 319 diarrheal stool samples in this study, which is relatively similar to the result (86%) achieved in a previous research conducted in Khartoum state (Elhag et al., 2013). These patients are likely infected by other pathogens not tested in both studies such as Sapovirus, Hepatitis A virus, *Staphylococcus aureus*, Aeromonas, other serotypes of *E. coli*, and parasites. Therefore, further research should be attempted to target these pathogens.

## Conclusion

In conclusion, this is the first comprehensive report to elaborate commonly known pathogen spectrum associated with childhood diarrhea in Khartoum State, Sudan. The results obtained in the present study highlight urgency of taking appropriate intervention measures in Khartoum State, Sudan.

## Author contributions

MA, IK, and XM designed the experiment; MA, JW, and HS did the lab experiment; HW and AM analyzed the data; MA, K-AE, and AE collected the samples. MA, JW, and AE wrote the article.

### Conflict of interest statement

The authors declare that the research was conducted in the absence of any commercial or financial relationships that could be construed as a potential conflict of interest.
