# Supplementary material for: Molecular Survey of Viral and Bacterial Causes of Childhood Diarrhea in Khartoum State, Sudan
Source: Front Microbiol. 2018 Feb 12;9:112. doi: 10.3389/fmicb.2018.00112 (PMC5816574; doi:10.3389/fmicb.2018.00112)
Supplement: Supplementary file 1 [file DataSheet1.DOC]

>Adv-Sample 28

AGGCAGCGCCCAACAGACCAAACTACATTGGCTTTAGGGATAACTTCATTGGCCTGATGT

ACTACAACTCCACAGGCAATATGGGCGTATTGGCTGGGCAGGCTTCACAGCTAAATGCTG

TAGTGGACTTGCAAGACAGGAACACTGAGTTATCGTACCAACTTATGCTGGATGCTCTTG

GCGATCGGAGCAGATATTTTTCCATGTGGAATCAGGCTGTTGACAGTTACGACCCCGACG

TAAGAATCATTGAGAACCACGGAGTGGAGGACGAAATGCCAAATTACTGCTTTCC

>Adv-Sample 217

AGGCAGCGCCCAACAGACCGAACTACATTGGCTTTAGGGATAACTTCATCGGCCTGATGT

ACTACAACTCCACAGGCAATATGGGTGTATTGGCTGGGCAAGCTTCACAGCTAAATGCTG

TAGTGGACTTGCAAGACAGGAACACTGAGTTATCATACCAACTTATGCTGGACGCACTTG

GCGATCGGAGCAGATATTTTTCTATGTGGAATCAGGCTGTTGACAGTTACGACCCCGACG

TAAGGATCATTGAGAACCACGGAGTGGAGGACGAAATGCCAAATTACTGCTTTCC

>Adv-Sample 196

AGGCAGCGCCCAACAGACCAAACTACATTGGCTTTAGGGATAACTTCATTGGCCTGATGT

ACTACAACTCCACAGGCAATATGGGCGTATTGGCTGGGCAGGCTTCACAGCTAAATGCTG

TAGTGGACTTGCAAGACAGGAACACTGAGTTATCGTACCAACTTATGCTGGATGCTCTTG

GCGATCGGAGCAGATATTTTTCCATGTGGAATCAGGCTGTTGACAGTTACGACCCCGACG

TAAGAATCATTGAGAACCACGGAGTGGAGGACGAAATGCCAAATTACTGCTTTCC

>Adv-Sample 250

AGGCAGCGCCCAACAGACCGAACTACATTGGCTTTAGGGATAACTTCATCGGCCTGATGT

ACTACAACTCCACAGGCAATATGGGTGTATTGGCTGGGCAAGCTTCACAGCTAAATGCTG

TAGTGGACTTGCAAGACAGGAACACTGAGTTATCATACCAACTTATGCTGGACGCACTTG

GCGATCGGAGCAGATATTTTTCTATGTGGAATCAGGCTGTTGACAGTTACGACCCCGACG

TAAGGATCATTGAGAACCACGGAGTGGAGGACGAAATGCCAAATTACTGCTTTCC

>Adv-Sample 299

AGGCAGCGCCCAACAGACCGAACTACATTGGCTTTAGGGATAACTTCATCGGCCTGATGT

ACTACAACTCCACAGGCAATATGGGTGTATTGGCTGGGCAAGCTTCACAGCTAAATGCTG

TAGTGGACTTGCAAGACAGGAACACTGAGTTATCATACCAACTTATGCTGGACGCACTTG

GCGATCGGAGCAGATATTTTTCTATGTGGAATCAGGCTGTTGACAGTTACGACCCCGACG

TAAGGATCATTGAGAACCACGGAGTGGAGGACGAAATGCCAAATTACTGCTTTCC

>Adv-Sample 332

AGGCAGCGCCCAACAGACCGAACTACATTGGCTTTAGGGATAACTTCATCGGCCTGATGT

ACTACAACTCCACAGGCAATATGGGTGTATTGGCTGGGCAAGCTTCACAGCTAAATGCTG

TAGTGGACTTGCAAGACAGGAACACTGAGTTATCATACCAACTTATGCTGGACGCACTTG

GCGATCGGAGCAGATATTTTTCTATGTGGAATCAGGCTGTTGACAGTTACGACCCCGACG

TAAGGATCATTGAGAACCACGGAGTGGAGGACGAAATGCCAAATTACTGCTTTCC

>KP274041/type F/Adenovirus 41

AGGCAGCGCCCAACAGACCAAACTACATTGGCTTTAGGGATAACTTCATTGGCCTGATGT

ACTACAACTCCACAGGCAATATGGGCGTATTGGCTGGGCAGGCTTCACAGCTAAATGCTG

TAGTGGACTTGCAAGACAGGAACACTGAGTTATCGTACCAACTTATGCTGGATGCTCTTG

GCGATCGGAGCAGATATTTTTCCATGTGGAATCAGGCTGTTGACAGTTACGACCCCGACG

TAAGAATCATTGAGAACCACGGAGTGGAGGACGAATTGCCAAATTACTGCTTTCC

>AB610520/type F/Adenovirus 41

AGGCAGCGCCCAACAGACCAAACTACATTGGCTTTAGGGATAACTTCATTGGCCTGATGT

ACTACAACTCCACAGGCAATATGGGCGTATTGGCTGGGCAGGCTTCACAGCTAAATGCTG

TAGTGGACTTGCAAGACAGGAACACTGAGTTATCGTACCAACTTATGCTGGATGCTCTTG

GCGATCGGAGCAGATATTTTTCCATGTGGAATCAGGCTGTTGACAGTTACGACCCCGACG

TAAGAATCATTGAGAACCACGGAGTGGAGGACGAATTGCCGAATTACTGCTTTCC

>KF303071/type F/Adenovirus 41

AGGCAGCGCCCAACAGACCAAACTACATTGGCTTTAGGGATAACTTCATTGGCCTGATGT

ACTACAACTCCACAGGCAATATGGGCGTATTGGCTGGGCAGGCTTCACAGCTAAATGCTG

TAGTGGACTTGCAAGACAGGAACACTGAGTTATCGTACCAACTTATGCTGGATGCTCTTG

GCGATCGGAGCAGATATTTTTCCATGTGGAATCAGGCTGTTGACAGTTACGACCCCGACG

TAAGAATCATTGAGAACCACGGAGTGGAGGACGAATTGCCAAATTACTGCTTTCC

>AB728839/type F/Adenovirus 41

AGGCAGCGCCCAACAGACCAAACTACATTGGCTTTAGGGATAACTTCATTGGCCTGATGT

ACTACAACTCCACAGGCAATATGGGCGTATTGGCTGGGCAGGCTTCACAGCTAAATGCTG

TAGTGGACTTGCAAGACAGGAACACTGAGTTATCGTACCAACTTATGCTGGATGCTCTTG

GCGATCGGAGCAGATATTTTTCCATGTGGAATCAGGCTGTTGACAGTTACGACCCCGACG

TAAGAATCATTGAGAACCACGGAGTGGAGGACGAATTGCCAAATTACTGCTTTCC

>DQ315364/type F/Adenovirus 41

AGGCAGCGCCCAACAGACCGAACTACATTGGCTTTAGGGATAACTTTATCGGCCTGATGT

ACTACAACTCCACAGGCAATATGGGTGTATTGGCTGGGCAAGCTTCACAGCTAAATGCTG

TAGTGGACTTGCAAGACAGGAACACTGAGTTATCATACCAACTTATGCTGGACGCACTTG

GCGATCGGAGCAGATATTTTTCTATGTGGAATCAGGCTGTTGACAGTTACGACCCCGACG

TAAGGATCATTGAGAACCACGGAGTGGAGGACGAACTGCCAAATTACTGCTTTCC

>HQ005287/type F/Adenovirus 41

AGGCAGCGCCCAACAGACCAAACTACATTGGCTTTAGGGATAACTTCATTGGCCTGATGT

ACTACAACTCCACAGGCAATATGGGCGTATTGGCTGGGCAGGCTTCACAGCTAAATGCTG

TAGTGGACTTGCAAGACAGGAACACTGAGTTATCGTACCAACTTATGCTGGATGCTCTTG

GCGATCGGAGCAGATATTTTTCCATGTGGAATCAGGCTGTTGACAGTTACGACCCCGACG

TAAGAATCATTGAGAACCACGGAGTGGAGGACGAATTGCCAAATTACTGCTTTCC

>AB103344/type F/Adenovirus 41

AGGCAGCGCCCAACAGACCGAACTACATTGGCTTTAGGGATAACTTCATTGGCCTGATGT

ACTACAACTCCACAGGCAATATGGGCGTATTGGCTGGGCAGGCTTCACAGCTAAATGCTG

TAGTGGACTTGCAAGACAGGAACACTGAGTTATCGTACCAACTTATGCTGGATGCTCTTG

GCGATCGGAGCAGATATTTTTCCATGTGGAATCAGGCTGTTGACAGTTACGACCCCGACG

TAAGAATCATTGAGAACCACGGAGTGGAGGACGAATTGCCAAATTACTGCTTTCC

>EF429127/type F/Adenovirus 41

AGGCAGCGCCCAACAGACCGAACTACATTGGCTTTAGGGATAACTTCATTGGCCTGATGT

ACTACAACTCCACAGGCAATATGGGCGTATTGGCTGGGCAGGCTTCACAGCTAAATGCTG

TAGTGGACTTGCAAGACAGGAACACTGAGTTATCGTACCAACTTATGCTGGATGCTCTTG

GCGATCGGAGCAGATATTTTTCCATGTGGAATCAGGCTGTTGACAGTTACGACCCCGACG

TAAGAATCATTGAGAACCACGGAGTGGAGGACGAATTGCCAAATTACTGCTTTCC

>NC_010956/type D/Adenovirus 9

AGTCCATGCCAAACAGGCCCAACTACATCGGCTTCAGGGACAACTTTGTGGGTCTCATGT

ACTATAACAGCACTGGCAACATGGGTGTGCTGGCTGGTCAAGCATCTCAGTTGAATGCTG

TGGTCGACTTGCAAGACAGAAACACAGAGCTGTCTTACCAGCTCTTGCTAGATTCTCTGG

GTGACAGAACCAGATACTTTAGCATGTGGAACTCTGCAGTGGACAGTTATGATCCTGATG

TCAGGATTATTGAAAATCACGGTGTGGAAGATGAACTTCCAAACTATTGCTTCCC

>AC_000006/type D/Adenovirus 17

AGTCCATGCCCAACAGGCCTAACTACATTGGCTTCAGAGACAACTTTGTGGGACTCATGTACTACAACAGTACTGGCAACATGGGTGTGCTGGCTGGTCAGGCCTCTCAATTGAATGCTGTGGTCGACTTGCAAGACAGAAACACCGAGCTGTCTTACCAGCTCTTGCTAGATTCTCTGGGTGACAGAACCAGATACTTCAGCATGTGGAACTCTGCGGTGGATAGCTATGATCCAGATGTCAGGATCATTGAAAATCATGGTGTGGAAGATGAACTTCCAAACTATTGCTTCCC

>AY487947/type E/Adenovirus 4

AGGCCATGCCTAACAGACCCAACTACATTGGCTTCAGAGACAATTTTATCGGGCTCATGTACTACAACAGCACTGGCAATATGGGGGTGCTGGCCGGTCAGGCCTCTCAGCTGAATGCTGTGGTTGACTTGCAAGACAGAAACACTGAACTGTCCTACCAGCTCTTGCTTGACTCTCTGGGTGACAGAACCCGGTATTTCAGTATGTGGAATCAGGCGGTGGACAGCTATGATCCTGATGTGCGCATTATTGAAAACCATGGTGTGGAGGATGAATTGCCAAACTATTGCTTTCC

>NC_001460/type A/Adenovirus 12

AAGCAGCGCCAAATAGAGCAAACTACATTGCATTCAGAGATAATTTTATTGGCCTGATGTATTACAACAGTACAGGCAACATGGGTGTATTGGCCGGGCAAGCTTCCCAACTTAACGCAGTAGTAGACCTGCAAGACAGAAATACAGAGCTGTCATACCAGTTAATGCTGGATGCTTTGGGAGACAGAACACGGTACTTTTCCTTGTGGAATTCCGCAGTGGACAGTTACGACCCTGACGTTCGCGTTATTGAGAATCACGGGGTAGAGGATGAACTACCAAATTATTGCTTTCC

>DQ149611/type A/Adenovirus 31

AAGCAGCACCTAACAGACCAAATTACATTGCCTTCAGAGACAATTTTATTGGGCTTATGTATTACAACAGCACTGGAAACATGGGAGTACTTGCAGGACAGGCTTCTCAGCTCAATGCAGTGGTTGATCTGCAAGATAGGAACACAGAGCTGTCCTACCAACTTATGTTGGATGCTTTAGGCGATAGAAGCCGTTATTTTTCCATGTGGAATTCTGCCGTCGACAGCTACGATCCAGACGTGCGAATTATTGAAAATCATGGTGTGGAAGACGAACTGCCCAATTACTGTTTTCC

>DQ149610/type A/Adenovirus 18

AAGCAGCGCCTAACAGACCCAATTACATTGGCTTCAGAGATAACTTCATAGGACTAATGTACTACAACAGCACTGGAAATATGGGAGTGCTTGCCGGACAGGCTTCGCAGCTTAATGCAGTGGTGGACCTGCAGGACAGGAATACAGAGCTGTCCTACCAACTTATGCTAGACGCTTTAGGCGACCGCAGTCGGTATTTTTCTATGTGGAACTCAGCCGTGGACAGCTACGATCCTGATGTGCGAATTATTGAAAATCATGGGGTGGAAGACGAACTTCCCAATTACTGCTTTCC

>DQ923122/type G/Adenovirus 52

AAGCCGCTCCAAACCGTCCAAATTACATTGCTTTTAGGGACAATTTTATTGGACTAATGTATTACAACAGCACGGGAAACATGGGGGTGCTTGCCGGTCAGGCTTCGCAGCTAAATGCGGTAGTAGATTTGCAAGATAGAAATACGGAACTATCCTACCAGCTGATGTTGGATGCACTGGGAGACAGAAGTCGCTACTTTTCTATGTGGAATCAAGCCGTAGATAGTTATGATCCAGATGTTAGAATTGTGGAAAATCACGGAGTAGAGGACGAACTACCAAACTATTGTTTCCC

>AY598970/type B/Adenovirus 11

AATCTATGCCCAACAGACCCAACTACATTGGCTTCAGAGATAACTTTATTGGACTTATGTACTATAACAGTACTGGTAACATGGGGGTGCTGGCTGGTCAAGCGTCTCAGTTAAATGCAGTGGTTGACTTGCAGGACAGAAACACAGAACTTTCTTACCAACTCTTGCTTGACTCTCTGGGCGACAGAACCAGATACTTTAGCATGTGGAATCAGGCTGTGGACAGTTATGATCCTGATGTACGTGTTATTGAAAATCATGGTGTGGAAGATGAACTTCCCAACTACTGTTTTCC

>AC_000018/type B/Adenovirus 7

AAGCCATGCCTAACAGACCCAATTACATTGGCTTCAGGGATAACTTTGTAGGTCTTATGTACTACAACAGTACTGGAAATATGGGAGTTTTGGCCGGCCAAGCATCACAACTGAATGCAGTGGTTGACTTGCAGGACAGAAACACTGAACTGTCATATCAGCTTTTGCTTGATTCTCTGGGAGACAGAAGCAGATACTTCAGCATGTGGAATCAGGCTGTGGACAGCTATGATCCCGATGTTCGTATTATTGAAAATCATGGCGTCGAGGATGAACTGCCTAATTACTGTTTTCC

>NC_001405/type C/Adenovirus 2

AATCTATGCCAAACAGACCCAATTACATTGCTTTCAGGGACAATTTTATTGGCCTAATGTATTATAACAGCACTGGCAACATGGGTGTTCTTGCTGGTCAGGCATCGCAGCTAAATGCCGTGGTAGATTTGCAAGACAGAAACACAGAGCTGTCCTATCAACTCTTGCTTGATTCCATAGGTGATAGAACCAGATATTTTTCTATGTGGAATCAGGCTGTAGACAGCTATGATCCAGATGTTAGAATCATTGAAAACCATGGAACTGAGGATGAATTGCCAAATTATTGTTTTCC

>AY339865/type C/Adenovirus 5

AATCTATGCCCAACAGGCCTAATTACATTGCTTTTAGGGACAATTTTATTGGTCTAATGTATTACAACAGCACGGGTAATATGGGTGTTCTGGCGGGCCAAGCATCGCAGTTGAATGCTGTTGTAGATTTGCAAGACAGAAACACAGAGCTTTCATACCAGCTTTTGCTTGATTCCATTGGTGATAGAACCAGGTACTTTTCTATGTGGAATCAGGCTGTTGACAGCTATGATCCAGATGTTAGAATTATTGAAAATCATGGAACTGAAGATGAACTTCCAAATTACTGCTTTCC

>AB330121/type F/Adenovirus 40

AGGCCGCTCCCAATCGACCTAATTACATTGGTTTTAGGGACAACTTCATTGGTTTGATGTACTACAATTCCACTGGCAACATGGGAGTGCTGGCCGGGCAAGCTTCTCAGCTCAACGCAGTGGTGGACTTACAAGATAGAAACACGGAGCTGTCTTACCAGTTAATGCTTGACGCTTTAGGGGATCGGAGTCGATACTTCTCCATGTGGAACCAGGCAGTGGACAGCTATGACCCAGACGTGAGAATTATTGAAAATCATGGCGTGGAAGACGAGCTCCCCAACTATTGCTTTCC
