# Supplementary material for: Molecular Survey of Viral and Bacterial Causes of Childhood Diarrhea in Khartoum State, Sudan
Source: Front Microbiol. 2018 Feb 12;9:112. doi: 10.3389/fmicb.2018.00112 (PMC5816574; doi:10.3389/fmicb.2018.00112)
Supplement: Supplementary file 2 [file DataSheet2.DOC]

>Boca- Sample 103

GGAGACATATGGATGTTTCCAAATCAAGTCTGGGACAGATATCCTATTACCAGAGAACATCCAATCTGGTGCAAAAAACCAAGGGCTGACAAACACACGATCATGGATCCATTTGATGGATCCATTGCAATGGATCATCCTCCAGGCACTATTTTTATAAAAATGGCAAAAATTCCAGTTCCAACTGCCTCAAATGCAGACTCATATCTAAACATATACTGCACTGGACAAGTCAGCTGTGAGATTGTATGGGAAGTAGAAAGATACGCAACAAAGAACTGGCGTCCAGAAAGAAGACATACTG

>Boca- Sample 21

GGGGACATATGGATGTTTCCTTATCGGATCTGGGACAGATTTCCTATCACCAGAGAAAATCCAATCTGGTGCAAAAAACCAAGGGCTGACAAACACACAATCATGGATCCATTTGATGGATCCATTGCAATGGATCATCCTCCAAGCACTATTTTTATAAAAATGGCAAAAATTCCAATTCCAACTGCAACAAATGCAAACTCATATCTAAACATATACTGTACTGAACAAGTCATCTGTGAAATTGTATGGGAGGAAGAAAAATACCCAACATACAACTGTCGTCCAGAAAGAACACATACTG

>DQ652181_NH2908

GGAGACATATGGATGTTTCCAAATCAAGTCTGGGACAGATTTCCTATCACCAGAGAACATCCAATCTGGTGCAAAAAACCAAGGGCTGACAAACACACAATCATGGATCCATTTGATGGATCCATTGCAATGGATCATCCTCCAGGCACTATTTTTATAAAAATGGCAAAAATTCCAGTTCCAACTGCCTCAAATGCAGACTCATACCTAAACATATACTGTACTGGACAAGTCAGCTGTGAGATTGTATGGGAAGTAGAAAGATACGCAACAAAGAACTGGCGTCCAGAAAGAAGACATACTG

>EF186869_Spain001

GGGGACATATGGATGTTTCCAAATCAAGTCTGGGACAGATTTCCTATCACCAGAGAAAATCCAATCTGGTGCAAAAAACCAAGAGCTGACAAACACACAATCATGGATCCATTTGATGGATCCATTGCAATGGATCATCCTCCAGGCACTATTTTTATAAAAATGGCAAAAATTCCAGTACCAACTGCAACAAATGCAGACTCATATTTAAACATATACTGTACTGGACAAGTCAGCTGTGAAATTGTATGGGAAGTAGAAAGATACGCAACAAAGAACTGGCGTCCAGAAAGAAGACATACTG

>EF186898_Spain030

GGGGACATATGGATGTTTCCAAATCAAGTCTGGGACAGATTTCCTATCACCAGAGAAAATCCAATCTGGTGCAAAAAACCAAGAGCTGACAAACACACAATCATGGATCCATTTGATGGATCAATTGCAATGGATCATCCTCCAGGCACTATTTTTATAAAAATGGCAAAAATTCCAGTTCCAACTGCCTCAAATGCAGACTCATACCTAAACATATACTGTACTGGACAAGTCAGCTGTGAGATTGTATGGGAAGTAGAAAGATACGCAACAAAGAACTGGCGTCCAGAAAGAAGACATACTG

>EF203921_CU49

GGGGACATATGGATGTTTCCAAATCAAGTCTGGGACAGATTTCCTATCACCAGAGAAAATCCAATCTGGTGCAAAAAACCAAGGGCTGACAAACACACAATCATGGATCCATTTGATGGATCCATTGCAATGGATCATCCTCCAGGCACTATTTTTATAAAAATGGCAAAAATTCCAGTACCAACTGCAACAAATGCAGACTCATATCTAAACATATACTGTACTGGACAAGTCAGCTGTGAGATTGTATGGGAAGTAGAAAGATACGCAACAAAGAACTGGCGCCCAGAAAGACGACATACTG

>EF584447_WLL-3

GGGGACATATGGATGTTTCCAAATCAAGTCTGGGACAGATTTCCTATCACCAGAGAAAATCCAATCTGGTGCAAAAAACCAAGGGCTGACAAACACACAATCATGGATCCATTTGATGGATCAATTGCAATGGATCATCCTCCAGGCACTATTTTTATAAAAATGGCGAAAATTCCAGTTCCAACTGCCTCAAATGCAGACTCATACCTAAACATATACTGTACTGGACAAGTCAGCTGTGAAATTGTATGGGAGGTAGAAAGATACGCAACAAAGAACTGGCGTCCAGAAAGAAGACATACTG

>EF450721_HK5

GGGGACATATGGATGTTTCCAAATCAAGTCTGGGACAGATTTCCTATCACCAGAGAAAATCCAATCTGGTGCAAAAAACCAAGAGCTGACAAACACACAATCATGGATCCATTTGATGGATCCATTGCAATGGATCATCCTCCAGGCACTATTTTTATAAAAATGGCAAAAATTCCAGTTCCAACTGCCTCAAATGCAGACTCATACCTAAACATATACTGTACTGGACAAGTCAGCTGTGAGATTGTATGGGAAGTAGAAAGATACGCAACAAAGAACTGGCGTCCAGAAAGAAGACATACTG

>EF450731_HK15

GGAGACATATGGATGTTTCCAAATCAAGTCTGGGACAGATTTCCTATCACCAGAGAACATCCAATCTGGTGCAAAAAACCAAGGGCTGACAAACACACAATCATGGATCCATTTGATGGATCCATTGCAATGGATCATCCTCCAGGCACTATTTTTATAAAAATGGCAAAAATTCCAGTTCCAACTGCCTCAAATGCAGACTCATACCTAAACATATACTGCACTGGACAAGTCAGCTGTGAGATTGTATGGGAAGTAGAAAGATACGCAACAAAGAACTGGCGTCCAGAAAGAAGACATACTG

>EF441542_MN869

GGGGACATATGGATGTTTCCAAATCAAGTCTGGGACAGATTTCCTATCACCAGAGAAAATCCAATCTGGTGCAAAAAACCAAGGACTGACAAACACACAATCATGGATCCATTTGATGGATCAATTGCAATGGATCATCCTCCAGGCACTATTTTTATAAAAATGGCAAAAATTCCAGTTCCAACTGCCTCAAATGCAGACTCATACCTAAACATATACTGTACTGGACAAGTCAGCTGTGAAATTGTATGGGAGGTAGAAAGATACGCAACAAAGAACTGGCGTCCAGAAAGAAGACATACTG

>EU069435_PEL/Ba/07

GGAGACATATGGATGTTTCCAAATCAAGTCTGGGACAGATTTCCTATCACCAGAGAAAATCCAATCTGGTGCAAAAAACCAAGGGCTGACAAACACACAATCATGGATCCATTTGATGGATCCATTGCAATGGATCATCCTCCAGGCACTATTTTTATAAAAATGGCAAAAATTCCAGTTCCAACTGCCTCAAATGCAGACTCATACCTAAACATATACTGTACTGGACAAGTCAGCTGTGAGATTGTATGGGAAGTAGAAAGATACGCAACAAAGAACTGGCGTCCAGAAAGAAGACATACTG

>EU370695_JL73

GGGGACATATGGATGTTTCCAAATCAAGTCTGGGACAGGTTTCCTATCACCAGAGAAAGTCCAATCTGGTGCAAAAAACCAAGAGCTGACAAACACACAATCATGGATCCATTTGATGGATCAATTGCAATGGATCATCCTCCAGGCACTATTTTTATAAAAATGGCAAAAATTCCAGTTCCAACTGCCTCAAATGCAGGCTCATACCTAAACATATACTGTACTGGACAAGTCAGCTGTGAAATTGTATGGGGGGTAGAAAGATACGCAACAAAGAACTGGCGTCCAGAAGGAAGACATACTG

>EU984233_TW2717_06

GGGGACATATGGATGTTTCCAAATCAAGTCTGGGACAGATTTCCTATCACCAGAGAACATCCAATCTGGTGCAAAAAACCAAGGGCTGACAAACACACAATCATGGATCCATTTGATGGATCCATTGCAATGGATCATCCTCCAGGCACTATTTTTATAAAAATGGCAAAAATTCCAGTTCCAACTGCCTCAAATGCAGACTCATACCTAAACATATACTGTACTGGACAAGTCAGCTGTGAGATTGTATGGGAAGTAGAAAGATACGCAACAAAGAACTGGCGTCCAGAAAGAAGACATACTG

>EU918736_W471

TCAGATGTATGGATGTTTCCAAATCAAATTTGGGACAGATACCCAGTAACAAGAGAAAATCCTATTTGGTGCAAACAACCAAGATCAGACAAACACACAACAATTGATCCTTTTGACGGATCAATAGCCATGGATCATCCACCAGGCACAATTTTCATCAAAATGGCAAAAATTCCAGTTCCTTCAAACAACAACGCAGACTCATACTTAAACATCTACTGCACTGGACAAGTCAGCTGCGAAATTGTCTGGGAAGTCGAAAGATATGCAACAAAGAACTGGAGACCAGAAAGAAGACACACAG

>FJ375129_SH3

TCAGATGTGTGGATGTTCCCAAATCAAATTTGGGACAGATATCCAATAACCAGAGAAAATCCAATCTGGTGCAAAAAACCAAGGTCAGACAAAAACACAATAATTGATCCTTTCGATGGAACACTTGCAATGGATCATCCTCCTGGAACGATCTTCATAAAAATGGCAAAAATTCCAGTTCCTTCAAACAACAACGCAGACTCATACCTAAACATCTACTGCACCGGACAAGTCAGCTGCGAAATTGTCTGGGAAGTTGAAAGATACGCAACAAAGAACTGGAGACCAGAAAGAAGACACACCG

>FJ973558_TU-A-114-06

TCAGATGTATGGATGTTCCCAAATCAAATTTGGGACAGATATCCAATAACCAGAGAAAATCCAATCTGGTGCAAAAAACCAAGATCAGATAAAAGCACAGTAATTGATCCTTTCGATGGAACACTCGCAATGGATCATCCTCCTGGAACAATCTTCATAAAAATGGCAAAAATTCCAGTTCCTTCAAACAACAACGCAGACTCATACCTAAACATCTACTGCACAGGACAAGTCAGCTGCGAAATTGTCTGGGAAGTTGAAAGATACGCAACAAAGAACTGGAGACCAGAGAGAAGACACACCG

>GQ891084_VRC60-IR

GGGGACATATGGATGTTTCCAAATCAAGTCTGGGACAGATTTCCTATCACCAGAGAAAATCCAATCTGGTGCAAAAAACCAAGGGCTGACAAACACACAATCATGGATCCATTTGATGGATCCATTGCAATGGATCATCCTCCAGGCACTATTTTTATAAAAATGGCAAAAATTCCAGTTCCAACTGCCTCAAATGCAGACTCATACCTAAACATATACTGCACTGGACAAGTCAGCTGTGAGATTGTATGGGAAGTAGAAAGATACGCAACAAAGAACTGGCGTCCAGAAAGAAGACATACTG

>GQ906592_GE4-IR

GGGGACATATGGATGTTTCCAAATCAAGTCTGGGACAGATTTCCTATCACCAGAGAAAATCCAATCTGGTGCAAAAAACCAAGGGCTGACAAACACACAATCATGGATCCATTTGATGGATCCATTGCAATGGATCATCCTCCAGGCACTATTTTTATAAAAATGGCAAAAATTCCAGTTCCAACTGCAACAAATGCAGACTCATATCTAAACATATACTGTACTGGACAAGTCAGCTGTGAAATTGTATGGGAAGTAGAAAGATACGCAACAAAGAACTGGCGTCCAGAAAGAAGACATACTG

>FJ973561_HBoV4-NI-385

TCAGATGTATGGATGTTTCCAAATCAAATTTGGGACAGATACCCAATAACCAGAGAAAATCCAATATGGTGTAAAAAACCCAGATCAGACAAACACACAACAATTGATCCTTTTGATGGATCCCTTGCAATGGATCATCCTCCAGGCACAATTTTTATTAAAATGGCAAAAATTCCAGTTCCTTCAAACAACAATGCAGACTCATACTTAAACATTTACTGCACAGGGCAAGTCAGCTGTGAAATTGTCTGGGAAGTTGAAAGATATGCAACAAAGAACTGGAGACCAGAAAGAAGACACACAA

>GU048662_CU47TH

TCAGATGTATGGATGTTCCCAAATCAAATTTGGGACAGATATCCAATAACCAGAGAAAATCCAATCTGGTGCAAAAAACCAAGATCAGATAAAAGCACAATAATTGATCCTTTTGATGGATCAATCGCAATGGATCATCCTCCTGGTACAATCTTCATAAAAATGGCAAAAATTCCAGTTCCTTCAAACAACAACGCAGACTCATACCTAAACATCTACTGCACAGGACAAGTCAGCTGCGAAATTGTCTGGGAAGTTGAAAGATACGCAACAAAGAACTGGAGACCAGAGAGAAGACACACCG

>GU563340_JP-8384

GGGGACATATGGATGTTTCCAAATCAAGTCTGGGACAGATTTCCTATCACCAGAGAAAATCCAATCTGGTGCAAAAAACCAAGGGCTGACAAACACACAATCATGGATCCATTTGATGGATCCATTGCAATGGATCATCCTCCAGGCACTATTTTTATAAAAATGGCAAAAATTCCAGTACCAACTGCAACAAATGCAGACTCATATCTAAACATATACTGTACTGGACAAGTCAGCTGTGAGATTGTATGGGAAGTAGAAAGATACGCAACAAAGAACTGGCGTCCAGAAAGAAGACATACTG

>JN794566_GZ9081

GGGGACATATGGATGTTTCCAAATCAAGTCTGGGACAGATATCCTATCACCAGAGAAAATCCAATCTGGTGCAAAAAACCAAGGGCTGACAAACACACAGTCATGGATCCATTTGATGGATCCATTGCAATGGATCATCCTCCAGGCACTATCTTTATAAAAATGGCAAAAATCCCGGTACCAACTGCCTCTAATGCAGACTCATATCTAAACATATACTGCACTGGACAAGTCAGCTGTGAAATTGTATGGGAGGTAGAAAGATACGCAACAAAGAACTGGCGTCCAGAAAGAAGACATACTG

>JN387085_CQ201012

GGGGACATATGGATGTTTCCAAATCAAGTCTGGGACAGATTTCCTATCACCAGAGAAAATCCAATCTGGTGCAAAAAACCAAGGGCTGACAAACACACAATCATGGATCCATTTGATGGATCAATTGCAATGGATCATCCTCCAGGCACTATCTTTATAAAAATGGCAAAAATCCCAGTACCAACTGCCTCTAATGCAGACTCATATCTAAACATATACTGCACTGGACAAGTCAGCTGTGAAATTGTATGGGAGGTAGAAAGATACGCAACAAAGAACTGGCGTCCAGAAAGAAGACATACTG

>JF272508_TJ155-2009

GGGGACATATGGATGTTTCCAAATCAAGTCTGGGACAGATTTCCTATCACCAGAGAAAATCCAATCTGGTGCAAAAAACCAAGGGCTGACAAACACACAATCATGGATCCATTTGATGGATCAATTGCAATGGATCATCCTCCAGGCACTATTTTTATAAAAATGACAAAAATTCCAGTTCCAACTGCCTCAAATGCAGACTCATACCTAAACATATACTGTACTGGACAAGTCAACTGTGAAATTGTATGGGAGGTACAAAGATACGCAACAAAGAACTGGCGTCCAGAAAGAAGACATACTG

>JQ411251_KU3

GGGGACATATGGATGTTTCCAAATCAAGTCTGGGACAGATTTCCTATCACCAGAGAAAATCCAATCTGGTGCAAAAAACCAAGAGCTGACAAACACACAATCATGGATCCATTTGATGGATCAATTGCAATGGATCATCCTCCAGGCACTATTTTTATAAAAATGGCAAAAATTCCAGTTCCAACTGCCTCAAATGCAGACTCATACCTAAACATATACTGTACTGGACAAGTCAGCTGTGAGATTGTATGGGAAGTAAAAAGATACGCAACAAAGAACTGGCGTCCAGAAAGAAGACATACTG

>JQ618264_Cam2009-P280

GGGGACATATGGATGTTTCCAAATCAAGTCTGGGACAGATTTCCTATCACCAGAGAACATCCAATCTGGTGCAAAAAACCAAGGGCTGACAAACACACAATCATGGATCCATTTGATGGATCCATTGCAATGGATCATCCTCCAGGCACTATCTTTATAAAAATGGCAAAAATTCCAGTACCAACTGCCTCTAATGCAGACTCATACCTAAACATATACTGTACTGGACAAGTCAGCTGTGAGATTGTATGGGAAGTAGAAAGATACGCAACAAAGAACTGGCGTCCAGAAAGAAGACATACTG

>JQ618275_Cam2008-E374

GGGGACATATGGATGTTTCCAAATCAAGTCTGGGACAGATTTCCTATCACCAGAGAACATCCAATCTGGTGCAAAAAACCAAGGGCTGACAAACACACAATCATGGATCCATTTGATGGATCCATTGCAATGGATCATCCTCCAGGCACTATCTTTATAAAAATGGCAAAAATTCCAGTACCAACTGCCTCTAATGCAGACTCATATCTAAACATATACTGCACTGGACAAGTCAGCTGTGAAATTGTATGGGAAGTAGAAAGATACGCAACAAAGAACTGGCGTCCAGAAAGAAGACATACTG

>JQ513498_Greece71.07

GGGGACATATGGATGTTTCCAAATCAAGTCTGGGACAGATTTCCTATCACCAGAGAAAATCCAATCTGGTGCAAAAAACCAAGGGCTGACAAACACACAATCATGGATCCATTTGATGGATCAATTGCAATGGATCATCCTCCAGGCACTATTTTTATAAAAATGGCAAAAATTCCAGTTCCAACTGCCTCAAATGCAGACTCATACCTAAACATATACTGTACTGGACAAGTTAGCTGTGAAATTGTATGGGAGGTAGAAAGATACGCAACAAAGAACTGGCGTCCAGAAAGAAGACATACTG

>JX887480_ZJ42

GGGGACATATGGATGTTTCCAAATCAAGTCTGGGGCAGATTTCCTATCACCAGAGAAAATCCAATCTGGTGCAAAAAACCAAGGGCTGACAAACACACAATCATGGATCCATTTGATGGATCAATTGCAATGGATCATCCTCCAGGCACTATTTTTATAAAAGTGGCAAAAATCCCAGTTCCAACTGCCTCAAATGCAGACTCATACCTAAACATATACTGTACTGGACAAGTTAGCTGTGAGATTGTATGGGAGGTAGAAAGATACGCAACAAAGAACTGGCGTCCAGAAAGAAGACATACTG

>KC544968_AR2011_2664

GGGGACATATGGATGTTTCCAAATCAAGTCTGGGACAGATTTCCTATCACCAGAGAACATCCAATCTGGTGCAAAAAACCAAGGGCTGACAAACACACAATCATGGATCCATTTGATGGATCAATTGCAATGGATCATCCTCCAGGCACTATTTTTATAAAAATGGCAAAAATTCCAGTTCCAACTGCAACAAATGCAGACTCATATCTAAACATATACTGTACTGGACAAGTCAGCTGTGAAATTGTATGGGAGGTAGAAAGATACGCAACAAAGAACTGGCGTCCAGAAAGAAGACATACTG

>KC544969_AR2011_2694

GGAGACATATGGATGTTTCCAAATCAAGTCTGGGACAGATTTCCTATCACCAGAGAAAATCCAATCTGGTGCAAAAAACCAAGGGCTGACAAACACACAATCATGGATCCATTTGATGGATCCATTGCAATGGATCATCCTCCAGGCACTATTTTTATAAAAATGGCAAAAATTCCAGTACCAACTGCAACAAATGCAGACTCATACCTAAACATATACTGTACTGGACAAGTCAGCTGTGAAATTGTATGGGAGGTAGAAAGATACGCAACAAAGAACTGGCGTCCAGAAAGAAGACATACTG

>DQ000495_ST1

GGGGACATATGGATGTTTCCAAATCAAGTCTGGGACAGATTTCCTATCACCAGAGAAAATCCAATCTGGTGCAAAAAACCAAGGGCTGACAAACACACAATCATGGATCCATTTGATGGATCCATTGCAATGGATCATCCTCCAGGCACTATTTTTATAAAAATGGCAAAAATTCCAGTACCAACTGCAACAAATGCAGACTCATATCTAAACATATACTGTACTGGACAAGTCAGCTGTGAAATTGTATGGGAAGTAGAAAGATACGCAACAAAGAACTGGCGTCCAGAAAGAAGACATACTG

>DQ000496_ST2

GGGGACATATGGATGTTTCCAAATCAAGTCTGGGACAGATTTCCTATCACCAGAGAAAATCCAATCTGGTGCAAAAAACCAAGGGCTGACAAACACACAATCATGGATCCATTTGATGGATCAATTGCAATGGATCATCCTCCAGGCACTATTTTTATAAAAATGGCAAAAATTCCAGTTCCAACTGCCTCAAATGCAGACTCATACCTAAACATATACTGTACTGGACAAGTCAGCTGTGAAATTGTATGGGAGGTAGAAAGATACGCAACAAAGAACTGGCGTCCAGAAAGAAGACATACTG

>KM257832_K2008001151

GGGGACATATGGATGTTTCCAAATCAAGTCTGGGACAGATTTCCTATCACCAGAGAAAATCCAATCTGGTGCAAAAAACCAAGGGCTGACAAACACACAATCATGGATCCATTTGATGGATCAATTGCAATGGATCATCCTCCAGGTACTATTTTTATAAAAATGGCAAAAATTCCAGTTCCAACTGCCTCAAATGCAGACTCATACCTAAACATATACTGTACTGGACAAGTCAGCTGTGAAATTGTATGGGAGGTAGAAAGATACGCAACAAAGAACTGGCGTCCAGAAAGAAGACATACTG

>KM257847_K2010001030

GGGGACATATGGATGTTTCCAAATCAAGTCTGGGACAGATTTCCTATCACCAGAGAAAATCCAATCTGGTGCAAAAAACCAAGGGCTGACAAACACACAATCATGGATCCATTTGATGGATCCATTGCAATGGATCATCCTCCAGGCACTATTTTTATAAAAATGGCAAAAATTCCAGTTCCAACTGCAACAAATGCAGACTCATATCTAAACATATACTGTACTGGACAAGTCAGCTGTGAAATTGTATGGGAGGTAGAAAGATACGCAACAAAGAACTGGCGTCCAGAAAGAAGACATACTG

>KM624025_LZFB080

TCAGATGTATGGATGTTCCCAAATCAAATTTGGGACAGATATCCAATAACCAGAGAAAATCCAATCTGGTGCAAAAAACCAAGATCAGATAAAAGCACAATAATTGATCCTTTTGATGGATCAATCGCAATGGATCATCCTCCTGGAACAATCTTCATAAAAATGGCAAAAATTCCAGTTCCTTCAAACAACAACGCAGACTCATACCTAAACATCTACTGCACAGGACAAGTCAGCTGCGAAATTGTCTGGGAAGTTGAAAGATACGCAACAAAGAACTGGAGACCAGAGAGAAGACACACCG
