# Supplementary material for: Molecular Survey of Viral and Bacterial Causes of Childhood Diarrhea in Khartoum State, Sudan
Source: Front Microbiol. 2018 Feb 12;9:112. doi: 10.3389/fmicb.2018.00112 (PMC5816574; doi:10.3389/fmicb.2018.00112)
Supplement: Supplementary file 3 [file DataSheet3.DOC]

**Accession number**  **Country Host** **Collection date** **Genotypes**  **Seq Id**

BankIt1873722 Seq1 KU220231 Sudan 05-sep-2014 Human Bocavirus1 103

BankIt1874849 Seq2 KU220232 Sudan 10-Aug-2014 Human Bocavirus1 21

BankIt1875076 Seq3 KU220233 Sudan 07-Agu-2014 type F/Adenovirus 41 28

BankIt1875076 Seq4 KU220234 Sudan 10-Sep-2014 type F/Adenovirus 41 217

BankIt1875076 Seq5 KU220235 Sudan 24-Sep-2014 type F/Adenovirus 41 196

BankIt1875076 Seq6 KU220236 Sudan 03-Oct-2014 type F/Adenovirus 41 250

BankIt1875076 Seq7 KU220237 Sudan 04-Nov-2014 type F/Adenovirus 41 299

BankIt1875076 Seq8 KU220238 Sudan 21-Jun-2014 type F/Adenovirus 41 332
