# Supplementary material for: Molecular Survey of Viral and Bacterial Causes of Childhood Diarrhea in Khartoum State, Sudan
Source: Front Microbiol. 2018 Feb 12;9:112. doi: 10.3389/fmicb.2018.00112 (PMC5816574; doi:10.3389/fmicb.2018.00112)
Supplement: Supplementary file 4 [file Image1.PDF]

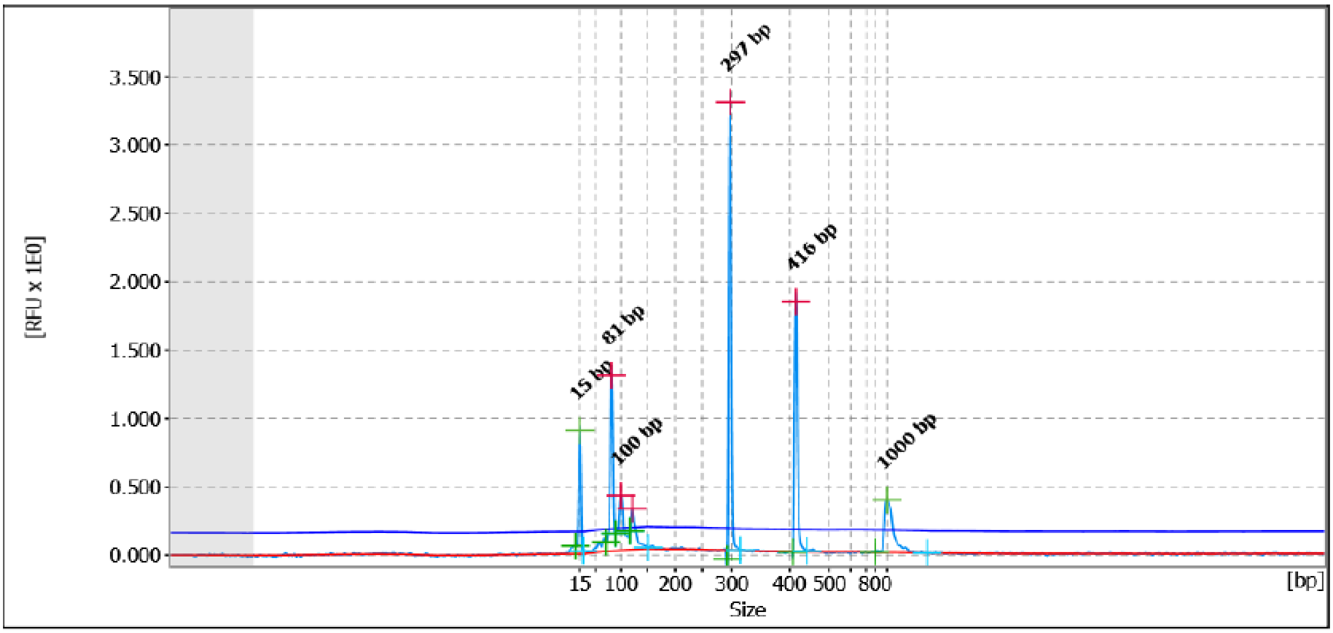

Figure 1: Electrophoresis results of tube 1 viral PCR products on automatic electrophoresis diagram explained the co-infection of sample number 103 with Noro virus G2 and human bocavirus. All of the Multiplex PCR viruses targets were identified successfully, and no mispriming was observed in tube 1 through using positive and negative control.

The red and blue line is the base line and threshold line, respectively. Any peak above blue line will be automatically calculated the fragment size.

15bp and 1000bp are the alignment markers that are used to align each run. Noro virus G2 (297 bp) and human bocavirus (418 bp).

Each peak will get a crossing above it, either green or red one. The green crossings are assigned to the peaks of alignment marker, while the red ones are for amplicon fragments.

The 81bp and 100bp are the products of unspecific amplification. We can tell the concentration of these products by their peak height. From this figure, we can see the peak of 297bp and 416bp are much higher than that of 81bp and 100bp, and so the amplifications of targets were not interfered by the unspecific amplifications.
