# Supplementary material for: Molecular Survey of Viral and Bacterial Causes of Childhood Diarrhea in Khartoum State, Sudan
Source: Front Microbiol. 2018 Feb 12;9:112. doi: 10.3389/fmicb.2018.00112 (PMC5816574; doi:10.3389/fmicb.2018.00112)
Supplement: Supplementary file 5 [file Image2.PDF]

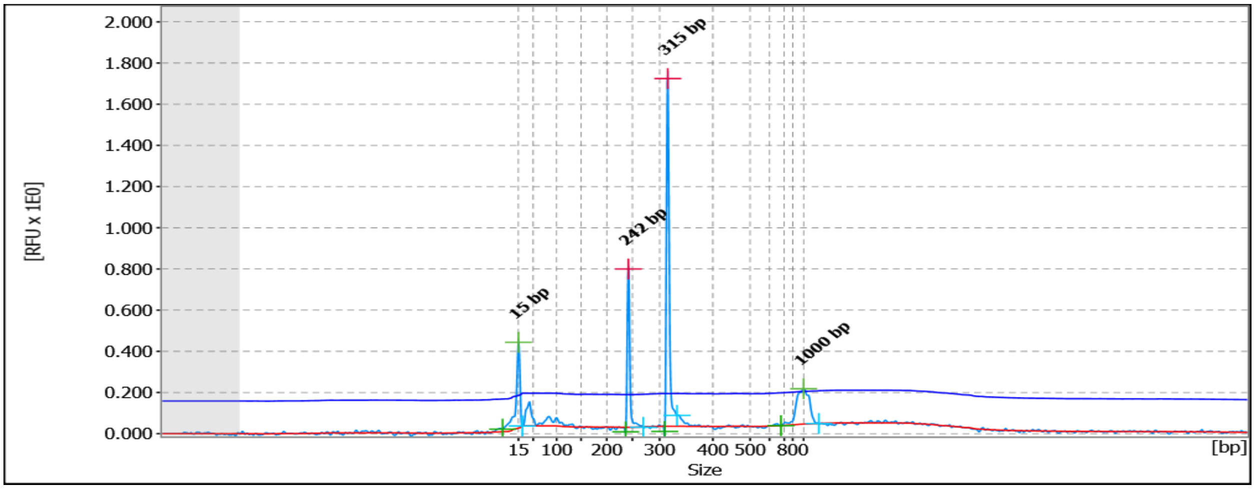

Figure 2 Electrophoresis diagram results of tube 2 bacterial Multiplex PCR products on automatic electrophoresis diagram explained co-infection of sample number 196 with *Vibrio parahaemolyticus* and *Salmonella enteritidis* . All of the targets were identified successfully, and no mispriming was observed in the tube 2 (through using positive and negative controls) marker (15 – 1000), *Vibrio parahaemolyticus*(242 bp) , *Salmonella enteritidis*(315 bp)

The red and blue line are the base line and threshold line, respectively. Any peak above blue line will be automatically calculated the fragment size.

15bp and 1000bp are the alignment markers that are used to align each run.

Each peak will get a crossing above it, either green or red one. The green crossings are assigned to the peaks of alignment marker, while the red ones are for amplicon fragments.
